# Effect of the implementation of a Birthing on Country service at a rural site, Waminda, compared to standard care for First Nations Australians: a prospective, non-randomised, interventional trial

**DOI:** 10.1016/j.lanwpc.2025.101796

**Published:** 2026-01-27

**Authors:** Yu Gao, Sue Kildea, Rebecca Coddington, Melanie Briggs, Cleone Wellington, Faye Worner, Donna Hartz, Juanita Sherwood, Yvette Roe

**Affiliations:** a Molly Wardaguga Institute for First Nations Birth Rights, Faculty of Health, Charles Darwin University, Casuarina, Northern Territory, 0810, Australia; b Waminda South Coast Women's Health and Wellbeing Aboriginal Corporation, Kinghorne Street, Nowra, New South Wales, 2541, Australia; c School of Nursing and Midwifery, Western Sydney University, Sydney, New South Wales, 2751, Australia; d Western Sydney Local Health District, North Parramatta, New South Wales, 2151, Australia; e Jumbunna Institute for Indigenous Education and Research (JIIER), University of Technology Sydney, Sydney, New South Wales, 2007, Australia

**Keywords:** Preterm, Birthing on Country, Wrap-around service, Birth

## Abstract

**Background:**

Clinical and cost-effectiveness of a Birthing on Country service has been demonstrated in a metropolitan centre. We sought to evaluate feasibility, clinical effectiveness and wrap-around supports in the rural setting by evaluating Waminda's Birthing on Country service.

**Methods:**

This prospective, non-randomised, interventional study was conducted in Nowra, Australia (ANZCTR: 12620000874910, study completed). Pre-defined primary outcomes were first assessment with health service in 1st trimester, ≥5 antenatal visits, normal birth, preterm birth, healthy baby and exclusive breastfeeding at discharge. Propensity score matching balanced confounders to calculate treatment effect. Waminda's wrap-around services and their interactions are represented using a network analysis.

**Findings:**

Relative to standard care, the Birthing on Country service was associated with significantly less women having ≥5 antenatal visits (80·6% versus 94·4%, odds ratio 0·22, 95% CI 0·10, 0·50) (with differences in measurements impacting this outcome), more normal births (32·8% versus 21·7%, odds ratio 1·77, 95% CI 1·08, 2·79), and exclusive breastfeeding at discharge (75·6% versus 63·3%, odds ratio 1·88, 95% CI 1·16, 3·05). No significant differences were observed in other primary outcomes. More than 90% of women accessing Waminda received at least one wrap-around service, some received intensive support.

**Interpretation:**

This study is the first to provide evidence towards successful implementation and effectiveness of a Birthing on Country service and the wrap around care in a rural setting and supports the urgent need for maternity service redesign for Aboriginal families.

**Funding:**

National Health Medical Research Council of Australia Partnership grant (grant 1135125).

**Research in context**

**Evidence before this study**

The Urban Demonstration Birthing on Country model was a successful partnership between a large maternity hospital and two Aboriginal Community Controlled Health Services. Combining continuity of midwifery carer with community-based Indigenous wrap around services and overarching Indigenous governance multiple non-clinical outcomes also improved. A reduced number of babies were removed from their mothers at birth, with increased Indigenous employment and education also documented. We hypothesised that women received assistance with housing, the justice system and family support services in culturally safe environment and this was impacting the outcomes. However, there was no evidence, to our knowledge, of what type of support was available, the uptake or the range of contacts that occur across the pregnancy journey.

**Added value of this study**

Waminda's Birthing on Country service improved clinical outcomes. Waminda's wrap-around programs focus on strength-based care addressing the social determinants of health impacting women's lives. This study, to the best of our knowledge for the first time, quantified the nature of cross-program supports in the Aboriginal community-controlled setting. More than 90% of women accessing Waminda received at least one wrap-around service and less women had interactions with the child safety system compared to women accessing standard care. Our results provide timely evidence towards successful implementation of a Birthing on Country service in a rural area for First Nations families.

**Implications of all the available evidence**

Waminda's Aboriginal Community-Controlled Birthing on Country service significantly improved outcomes when providing antenatal and postnatal continuity of care and wrap-around supports. Although not fully implemented, this builds on the evidence of the effectiveness of Birthing on Country service redesign and their feasibility in the rural setting, highlighting the comprehensiveness of the wrap around services. National implementation of Birthing on Country services should be supported by all levels of government to redress the ongoing harmful impacts of colonisation. Funding for maternity services should flow to the Aboriginal Community-Controlled sector.

## Introduction

For many thousands of years, Aboriginal women have used traditional birthing methods and medicines to provide holistic care for women during pregnancy, birth and the postpartum period.^1^ As the oldest continuous living culture on earth, Aboriginal Women possess undeniable strength, capacity and resilience. Since European colonisation, Aboriginal women's birthing wisdom and knowledge was and has been interrupted and prevented by western models of care.^1^^,^^2^ As a result of the detrimental impacts of colonisation, unacceptable maternal and infant health deaths and disparities exist for Aboriginal families and non-Aboriginal families in Australia^3^, ^4^, ^5^; with little or no improvement in key maternal child health (MCH) indicators for the past ten years. There is growing evidence that the chronic diseases (e.g., diabetes, hypertension, cardiovascular and renal disease) prevalent in adulthood for many Aboriginal peoples have their genesis in utero and early life as a result of colonial tenure limited access to healthy tucker and good health care.^6^^,^^7^ Preterm birth is the largest contributor to infant and child mortality^8^ and is associated with significant sequelae and lifelong inequity, including developmental and behavioural problems with negative impacts on schooling and educational attainment,^9^ childhood disability^8^ and chronic diseases in adulthood.^10^ Encouragingly, many risk factors for preterm birth,^11^ maternal mortality^12^ and other poor outcomes are modifiable and/or treatable. Aboriginal communities are coming forward with solutions to ‘Close the Gap’, challenging the colonisation of birth and seeking to restore birthing services back to community control.^1^^,^^12^^,^^13^

Since 2011, national maternity policy has recommended that Birthing on Country Services^14^ be established to provide culturally tailored maternity care. Birthing on Country is a mother and child centred way of providing the best start to life for Aboriginal babies with Aboriginal ways of knowing, being and doing, at the core of service design and delivery.^15^ Birthing On Country Services are considered a complex intervention with a suite of activities with multifaceted components that start in with pregnancy planning and continue across antenatal, intrapartum and the postnatal period with some extending into early childhood.^16^ Birthing support is provided by a known midwife with the location (home, hospital or birth centre) dependent on the woman and available local services. The conceptual framework for the Birthing on Country service has four interventional components: redesigning the health system to increase continuity of carer, investing in the workforce, strengthening the capacity of families and embedding community ownership.^17^ National guidelines for implementation and evaluation of Birthing on Country services,^15^ based on international evidence,^18^ and national consensus,^19^ were developed and endorsed by the Australian Health Ministers Advisory Council in 2013, with recommendations to establish exemplar services in urban, rural, remote and very remote areas. An urban demonstration service: the Birthing in Our Community (BiOC) service, in Meanjin (Brisbane), South East Queensland was established and evaluated through a prospective, non-randomised, interventional trial. It included: a multi-agency partnership between two Aboriginal Community-Controlled health services and a tertiary maternity hospital,^20^ a caseload midwifery group practice (MGP) working with an Indigenous workforce,^21^ Indigenous governance, a cultural safety framework, and a focus on integrating and coordinating care across primary and tertiary services.^22^ The study demonstrated that the women receiving BiOC care were more likely to attend five or more antenatal visits, less likely to have a preterm birth, and more likely to exclusively breastfeed on discharge from hospital^23^; and saved the Australian health system AUD 4810 per mother–baby pair.^24^ The model increased support for women, for example, with housing, financial assistance and interactions with the justice system, however, this was not quantified. Although small numbers, BiOC saw reduced removals of newborn babies compared to standard care (AOR 0·37, 95% CI 0·16, 0·84),^25^ an important finding, given Australia's rates of Aboriginal children in out of home care are twelve times the rate of non-Indigenous children.^26^

In 2017, a NHMRC partnership grant called Building on Our Strengths (BOOSt), was awarded to test translation by implementing and evaluating Birthing on Country Service models in two additional geographically diverse settings through a participatory action research (PAR) approach. The two sites were: The Institute for Urban Indigenous Health (IUIH) in Meanjin (Brisbane) Queensland (urban) and Waminda South Coast Women's Health and Wellbeing Aboriginal Corporation (hereafter Waminda) in Nowra, New South Wales (rural). This paper aims to report on the implementation (feasibility) and clinical effectiveness of the maternity service at Waminda, comparing key maternal and infant health outcomes with standard care, and to demonstrate the comprehensive nature of the wrap-around supports provided to Waminda clients. Further publications will report on the urban site and qualitative findings arising from the BOOSt study.

## Methods

### Study design and setting

This prospective, non-randomised, interventional study was conducted at Waminda, an Aboriginal Community Controlled Health Organisation located in Nowra, New South Wales (NSW), Australia, within the Illawarra Shoalhaven Local Health District. The Shoalhaven region is located on the unceded lands of the 13 Clans of the South Coast, about 2 h from Sydney by car.^27^

To better serve the healthcare needs of the local Aboriginal population, Waminda was established in 1984, originally known as ‘Jilimi’.^28^ In the late 1980s the organisation incorporated and evolved into the South Coast Women's Health and Welfare Corporation ‘Waminda’. Four decades later, Waminda remains an Aboriginal women-led organisation, guided by a board of seven Aboriginal women. Waminda provides comprehensive integrated primary health care clinics, maternity services, case management, and numerous health and wellbeing programs for Aboriginal women and their families within a strength-based framework. Waminda's Minga Gudjaga (Mother and Child) maternity service was established in 2011, offering continuity of services from pre-conception, antenatal and postnatal care for up to six weeks post birth to women having an Aboriginal baby within the local area. Waminda aimed to increase services to include intrapartum continuity of carer, however, multiple structural barriers meant this was not possible during the study timeframe,^29^ though they have since achieved this aim. At the time of this study, the Minga Gudjaga team consisted of 1·5 FTE of midwives, with three registered midwives sharing the provision of antenatal and postnatal care alongside their other responsibilities within Waminda. These included nursing, child and family health, and, for one team member, the Birthing on Country project officer work, which was needed to expand the existing model of care. Antenatal and postnatal care was provided either at the woman's home, or at Waminda's primary healthcare clinic in the regional centre of Nowra. The majority of women gave birth at the local public maternity hospital, Shoalhaven District Memorial Hospital or the Wollongong regional hospital. The key components of the Waminda Service and standard care available at the hospital are listed in Table 1 and detailed in the study protocol.^30^ The Minga Gudjaga program is supported by several internal wrap-around programs to prevent child removal. These include Nabu that provides casework for family preservation and restoration, Dead or Deadly focussing on holistic health promotion, exercise and nutrition and Balaang Gunyah Cultural Healing services, including healing counsellors etc. See Table 2 for a description of these services.

**Table 1:** Key components of Waminda service and standard care.

Waminda new model of care cohort (as it was 2018–22)	Standard care (reference cohort)
**Partnerships and governance**	
***Multi-agency Partnership Committee*** providing culturally responsive overarching governance and leadership of redesign of maternity services and research, community engagement in co-designing the new model of care.	No Aboriginal oversight of maternity services
**Continuity of carer across the maternity journey**	
Antenatal care is provided by Waminda's Minga Gudjaga maternity service within the Minga Gudjaga Gunyah clinic, the woman's home or hospital environment. Aboriginal Health Practitioners work with midwives to provide continuity of care, cultural and clinical support across the antenatal, and postnatal periods. Antenatal care using Aboriginal knowledges to address modifiable risk factors. Midwives follow the National Midwifery Guidelines for Consultation and Referral. When women are Categorised as B (midwives consult with the obstetric staff at the hospital or GP at Waminda, depending on the condition). When women are Category C, midwives consult with the specialist at the hospital to plan a specialist antenatal appointment and ongoing care at Shoalhaven District Memorial Hospital (SDMH) however women will continue to receive antenatal and postnatal continuity of care by their named midwife and receive specialist input as required (e.g., diabetes educator). Women have phone access to a known Waminda midwife during business hours. Outside of these hours, women were encouraged to contact the public maternity hospital. Minga Gudjaga Midwives collaborate with the O&G Consultant at the hospital. An Aboriginal Community Controlled Midwifery Group Practice that employs Endorsed Midwives to be established at Waminda to provide intrapartum care in hospital. *This was not established in the timeframe addressed in this paper.* After birth, when the woman and baby return to the community, the primary midwife and Waminda support services provide postnatal care for up to 6-weeks after which time Waminda's early childhood services continue to provide care. Waminda uses Communicare, which is a community-based system of formal clinical communication, which differs from the existing systems within the hospital. Written handover of care for transfer between services.	Antenatal care is provided at the hospital, by midwives, general practitioners or by the Aboriginal Maternal Infant Health Service teams. Aboriginal Maternal and Infant Health Service Aboriginal Health Workers provide community education support and care. They do not provide clinical care. Midwives at the hospital follow hospital guidelines. When women are receiving care from the GP they follow their own guidelines. No 24/7 phone access to a known (primary) midwife. Clinical care for women in labour is provided from a roster of midwives and may be a midwife the woman has never met. After birth, when the women and baby return to the community, postnatal care may be provided by the hospital or Waminda. Services communicate with referrals and discharge summaries via electronic mail (±phone). These are often missing or late. After birth, when women and baby return to the community they will receive postnatal care up to 2 weeks. After which time they hand over to child and family health service. Written handover of care for transfer between services.
**Aboriginal workforce**	
Women are referred to Waminda wrap around support services in line with guidelines. Aboriginal Health Practitioner embedded within the model will be the first point of contact within the maternity journey for all women. Transport officer for women who require assistance.	Women are referred to Aboriginal support services within the community, such as AMIHS and AMS.
**Cultural safety framework**	
Frontline staff are provided with clinical mentoring and reflective supervision with a focus on clinical and cultural safety, effective intercultural communication, and working together. The Wiyana Yanaga (Cultural Framework) is the foundation of Waminda. Informing this is the Waminda Cultural Committee, made up of local Aboriginal women who represent many family bloodlines who guide and inform all practices and protocols at Waminda. The Birthing on Country Manager provides cultural and operational leadership. Non-Aboriginal staff attend monthly ‘Imperfect Allies’ groups to support continual self-reflection of their White privilege to ensure they are working safely with Aboriginal staff and clients.	No ongoing provision of mentoring and reflective supervision.
**Holistic wrap around services integrating primary health care network with regional and tertiary services**	
Aboriginal women and their babies are offered cultural ceremonies after the birth of their baby. Support from Waminda programs, including family restoration and preservation services, diabetes educator, exercise physiologist, health and wellbeing services, healing programs, general case management and crisis support services (see network analysis and Table 2).	Aboriginal liaison officer and social worker available on referral.
**Community based hub** ± **birth centre**	
A community-based hub provides a culturally responsive and safe place for women, families, caregivers and Elders to undertake cultural activities that allow connection, sharing and learning from each other within a strength-based approach to birthing and parenting.	Some women have access to a community-based hub.

This table outlines the main differences between Waminda's new model of care (2018–2022) and the standard maternity care pathway, including governance structures, continuity of carer, Aboriginal workforce roles, cultural safety practices, communication processes, and access to wrap-around support services.

**Table 2:** Frequency of 185 Waminda women accessing Waminda's wrap-around service from 1st of pregnancy to 42 days after birth.

Program	Short description of the program	Number of women (%)	Mean number of visits ± SD	Range of visit
Domestic violence	High quality holistic support, advocacy and case management to women and their families who are victims of Family and/or Domestic Violence.	23 (12·4%)	14·8 ± 16·6	1–71
Balaang Healing	Yarning groups, short term accommodation, cultural mentoring and counselling for women on their healing journey led by Aboriginal women under guidance of Elders.	5 (2·7%)	2·6 ± 1·8	1–5
Child and parenting	Parents of children up to 12 years of age are eligible for one-to-one Parenting Programs via home visits covering practical parenting skills, and advocacy and liaison with other services such as NSW Department of Communities and Justice child protection.	23 (12·4%)	26·6 ± 35·5	1–127
Case management	Women facing multiple challenges in their lives are provided with a strength-based practice through working closely with partners within and outside of Waminda.	30 (16·2%)	15·2 ± 18·4	1–68
Aboriginal Health Practitioner (AHP)	Aboriginal Health Practitioner provides a range of clinical services (smoking cession support, social and emotional wellbeing, internal and external referrals, vaccination and annual health check) and advocacy with a focus on culturally safe care for Aboriginal people.	158 (85·4%)	4·9 ± 4·1	1–25
General Practitioner (GP)	General practitioners treat all common medical conditions and refer women to hospitals and other medical services for urgent and specialist treatment.	178 (96·2%)	13·6 ± 8·1	1–40
Registered nurse	Registered nurses offer vaccinations, sexual health screening, pathology collection, iron transfusions, dressings, healthcare advice and child and family health checks.	127 (68·6%)	2·9 ± 2·6	1–18
Social and emotional wellbeing	Women requiring Social and Emotional Wellbeing support are provided with holistic care including advocacy and referrals as needs are identified such as housing, health, employment, education, justice, mental health, detox, and rehabilitation services etc.	11 (5·9%)	22·1 ± 36·8	1–100
Drug and alcohol	Women with drug and alcohol addiction are provided with wrap-around services for detox, rehabilitation, transitioning back into community, court support and safety management plans.	12 (6·5%)	7·8 ± 16·0	1–58
Dead or deadly	The Dead or Deadly team emphasises and facilitates the importance of healthy lifestyles choices, using lifestyle medicine for chronic disease management and prevention, weight wellness, exercise, and smoking cessation to enhance the health, wellbeing and cultural connection of local Aboriginal women.	46 (24·9%)	9·5 ± 11·3	1–41
Allied health	Working with many teams across Waminda including playgroups, Nabu team and the healing counsellors etc., allied health professional such as dietician, optometrist, physiotherapist, podiatrist are available for women as needed.	19 (10·3%)	1·5 ± 0·8	1–4
Playgroup	Playgroups are an opportunity for young children to interact with other kids in a fun and safe environment, while giving parents and carers the opportunity to interact with other adults in a culturally safe environment.	10 (5·4%)	12·7 ± 18·9	1–47
Healing counsellor	Waminda's healing counsellors provide high quality therapeutic approaches to Aboriginal women and their families. The counselling team exists of: two Aboriginal Healing Counsellors, a Social and Emotional Wellbeing Counsellor, a Youth Counsellor and a Drug and Alcohol Counsellor.	37 (20·0%)	4·9 ± 4·8	1–20
Chronic care	The Integrated Team Care (ITC) program is aimed to support clients with management of their chronic disease/s. ITC aims to reduce hospital admissions, delay the progression of disease, and assist clients with understanding their medical conditions and ensuring good management of that condition.	6 (3·2%)	7·0 ± 4·4	2–12
Crisis support	Waminda's Intake program is the first point of contact for the service that provides immediate and crisis support to women.	44 (23·8%)	5·1 ± 4·0	1–17
Justice health	Justice Health supports women who come into contact with the Criminal Justice system. Waminda provide support both in the community and at correctional centres.	4 (2·2%)	12·8 ± 17·6	1–39
Child safety	Waminda provides family preservation and restoration program through intensive support, walking alongside Aboriginal families where the Department of Communities and Justice (DCJ) are involved with families.	28 (15·1%)	75·2 ± 90·3	1–373

This table summarises the proportion of 185 women who accessed each Waminda wrap-around program from the start of pregnancy to 42 days after birth. For each service, the number and percentage of women, mean number of visits (±SD), and visit range are reported.

Demographic data show that many members of the Aboriginal population in the study area experience socio-economic disadvantages with high unemployment rates, financial distress and homelessness. In 2021, the perinatal mortality rate for babies born to Aboriginal and Torres Strait Islander women (12·7 per 1000 births) was one and half times greater than the mortality rate experienced by their non-Indigenous counterparts (8·3 per 1000).^31^ Rates of smoking in pregnancy were high for Aboriginal mothers (39·9%) in Shoalhaven and the preterm birth rate was 10·4% versus 6·9% for non-Aboriginal women.^31^ During the study period, Aboriginal children in New South Wales were at eight times greater risk of having child protective service interaction than non-Indigenous children.^32^ In light of these outcomes, Waminda's service is designed to provide culturally safe care to women who are dealing with complex life stressors during the perinatal period, to support women and their Aboriginal families to not just survive, but to thrive.

### Procedure and participants

At Waminda, women carrying an Aboriginal baby could either self-refer, or be referred to the Minga Gudjaga team by any healthcare provider. Pregnant women would commonly see a Waminda Aboriginal Health Practitioner and General Practitioner (GP) to confirm their pregnancy and then referred to the Minga Gudjaga clinic where their antenatal care would be continued with a midwife. Women who had previously received care from Minga Gudjaga commonly self-referred by contacting their known midwife once pregnant. Women were eligible for care with Minga Gudjaga if they were carrying an Aboriginal baby (i.e., either the mother or father of the baby is Aboriginal) and were residing within the Shoalhaven region. Antenatal and postnatal midwifery care was provided during business hours, Monday to Friday, with no formal on-call component of care. If a Minga Gudjaga client went into labour during business hours, a Waminda midwife may attend the hospital as a support person, if her workload allowed and with the woman's consent.

Women carrying an Aboriginal baby who did not receive Waminda maternity service were defined as the standard care cohort. Randomisation of participants had been discussed prior to applying for research funding however key leaders within Waminda preferred a non-randomised prospective trial design, much the same as the Brisbane demonstration service redesign.^23^

### Data sources

**Fig. 1:**
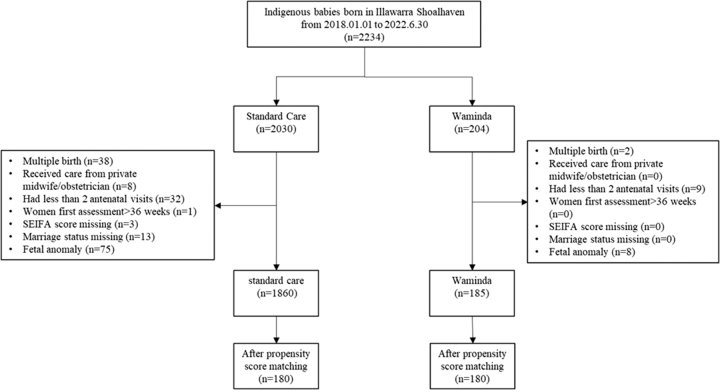
**Study participants’ flowchart.** This flow chart summarises the progression of participants through each stage of the study, including initial eligibility screening, exclusions (with reasons), and final numbers included in the analysis. All counts represent unique participants.

In total, 2234 birth records of all Aboriginal babies born at the Shoalhaven District Memorial Hospital and the Wollongong Hospital between 1st January 2018 and 30th June 2022 were extracted for this prospective trial from the hospital's routinely collected and contemporaneously entered obstetric database eMaternity. A total of 189 records were excluded following the pre-defined exclusion criteria^30^ and 2045 women carrying a singleton Aboriginal baby were included in analysis: 1860 received standard care and 185 received Waminda care (Fig. 1).

The frequency of the wrap-around services contacts for the Waminda clients, between 1st day of pregnancy and 42 days after birth, were extracted from Waminda's electronic health record system Communicare. Twenty-eight relevant wrap-around programs were identified, and some programs were grouped into a broader category when the contacts were fewer than four. In total, 17 wrap-around services were included in the final analysis.

### Outcomes

All primary and secondary outcomes reported were predefined. Primary maternal outcomes were: first antenatal assessment with health service in the 1st trimester (<14 weeks), number of antenatal visits (less than 5 or ≥5), normal birth (≥37 weeks, vertex presentation, spontaneous onset of labour, no analgesia, spontaneous vaginal birth, no episiotomy). Primary infant outcomes were: preterm birth (>20 weeks or 400 g and <37 weeks), healthy baby (live born, singleton, ≥37 weeks, 2500–4499 g, Apgar score at 5 min ≥ 7) and exclusive breastfeeding at discharge.^30^ Secondary outcomes included spontaneous labour onset, induction of labour, epidural pain relief, spontaneous vaginal birth, caesarean section, 3rd or 4th degree perineal tear, episiotomy, low birthweight (<2500 g), and Apgar score less than 7 at 5 min.

### Confounders

In line with the best practice we have adjusted for confounders in the propensity score matching analysis that are measured at baseline and not post-baseline covariates that may be influenced or modified by the model of care.^33^ The following confounders at baseline were adjusted: mother's Indigenous status, teenager (maternal age under 20 years) status, Social-Economic Indexes for Areas (SEIFA) quintile, marriage status, body mass index, primiparity, previous caesarean section, previous preterm, medical history of heart disease, diabetes, thyroid disease, liver disease, haematological disease, hypertension, renal disease, and sexually transmitted infection, mental health status at booking, smoking and illicit drug usage at booking. Pregnancy complications developed after 20 weeks gestation are treated as outcomes as they are influenced by the model of care, not baseline confounders.

### Ethics

Ethics approval was granted by the Aboriginal Health & Medical Research Council (AH&MRC, NSW) Ethics Committee (1448/18, 5 December 2018), Mater Human Research Ethics Committee (HREC/18/MHS/107, 7 September 2018), University of Queensland Human Research Ethics (2018001973/HREC/18/MHS/107, 28 September 2018), Charles Darwin University Human Research Ethics Committee (H19054, 10 July 2019) and UOW & ISLHD Health and Medical Human Research Ethics Committee (2018/472, 18 December 2018). Waiver of consent was approved by HREC for all women's routinely collected clinical data recorded in the hospital and Waminda. The trial was registered at the Australia & New Zealand Clinical Trial Registry #ACTRN12620000874910 (2 September 2020). Our research are aligned with and guided by the CONSIDER statement^34^ and STROBE reporting guidelines.^35^

### Statistical analysis

Number and frequency are presented for categorical variables, medians and interquartile ranges (IQR) for non-normally distributed continuous variables and mean and standard deviation for normally distributed continuous variables.

**Fig. 2:**
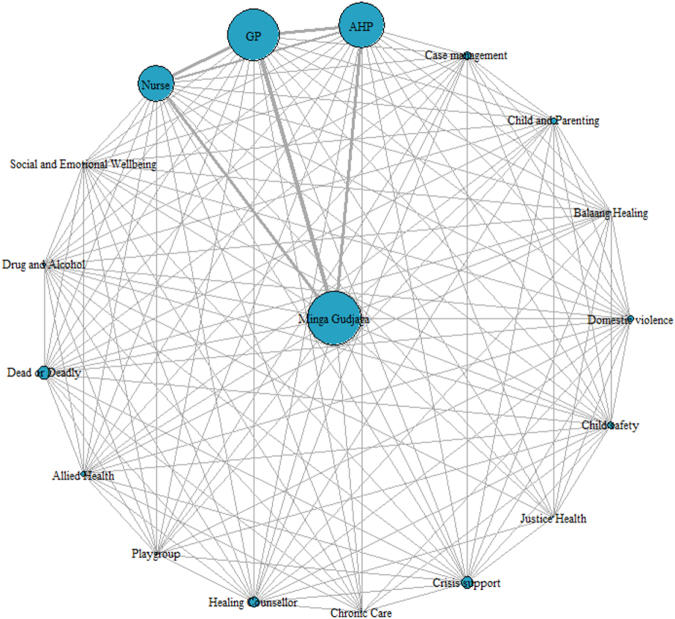
**Network analysis displaying women's interactions with the midwifery service and Waminda's Wrap-around services.** This network diagram illustrates how women engaged with Minga Gudjaga (Waminda's Mother and Child maternity service) and a broad range of programs. Node size represents the number of women participating in each service, while edge thickness reflects the frequency of movements or shared engagement between services. Key programs include Dead or Deadly (Waminda's chronic disease prevention and exercise program) and Balaang Healing (a healing group for young women). The diagram highlights the central coordinating role of Minga Gudjaga and its strong connections with clinical providers, including GP, AHP, and nursing services.

To represent the wrap-around services and their interactions, we constructed a network structure (Fig. 2) with nodes representing the programs, and edges representing contacts between programs. The node size is proportional to the program participants or engagement, and the edge thickness is proportional to the frequency of interaction. The connection between programs was unidirectional, as referral-in or referral-out sources were not recorded. The network visualisation was conducted using R version 4·1·1, and igraph package version 2·0·3.^36^

We performed propensity score matching analyses to account for confounding bias in estimating the treatment effects using observational data. All baseline characteristics described in the confounders section were adjusted for (Table 3).

**Table 3:** Maternal characteristics comparisons among two cohorts women who gave birth to an Indigenous baby during 01/01/2018–30/06/2022.

	Before matching			After matching		
	Standard care	Waminda	Bias	Standard care	Waminda	Bias
	(n = 1860)	(n = 185)	(%)	(n = 180)	(n = 180)	(%)
Indigenous mum	1008 (54·2%)	147 (79·5%)	55·1	138 (76·7%)	142 (78·9%)	4·9
Teenager (maternal age <20 years)	147 (7·9%)	27 (14·6%)	21·1	24 (13·3%)	27 (15·0%)	5·3
Socioeconomic status (SEIFA)						
Quintile 1 (most disadvantaged)	688 (37·0%)	118 (63·8%)	54·6	112 (62·2%)	113 (62·8%)	1·2
Quintile 2	652 (35·1%)	64 (34·6%)	−1·8	68 (37·8%)	64 (35·6%)	−4·7
Quintile 3	342 (18·4%)	2 (1·1%)	−61·5	0 (0·0%)	2 (1·1%)	3·9
Quintile 4	157 (8·4%)	1 (0·5%)	−39·1	0 (0·0%)	1 (0·6%)	2·7
Quintile 5 (most advantaged)	21 (1·1%)	0 (0·0%)	–	0 (0·0%)	0 (0·0%)	–
Married or in de facto	1064 (57·2%)	47 (25·4%)	−67·7	48 (26·7%)	47 (26·1%)	−1·2
BMI, median (IQR)	25·0 (21·5, 30·8)	24·4 (21·1, 28·0)	−20·7	24·6 (20·6, 30·3)	24·5 (21·2, 28·3)	−5·3
Primiparity	688 (37·0%)	73 (39·5%)	5·0	68 (37·8%)	72 (40·0%)	4·6
Previous caesarean section	345 (18·5%)	25 (13·5%)	−14·0	29 (16·1%)	25 (13·9%)	−6·1
Previous preterm birth	201 (10·8%)	21 (11·4%)	1·3	19 (10·6%)	19 (10·6%)	1·0
Medical history of Heart disease	93 (5·0%)	12 (6·5%)	6·1	11 (6·1%)	11 (6·1%)	0·0
Medical history of diabetes (Type 1 or 2)	15 (0·8%)	1 (0·5%)	−3·3	1 (0·6%)	1 (0·6%)	0·0
Medical history of thyroid disease	96 (5·2%)	11 (5·9%)	3·4	13 (7·2%)	9 (5·0%)	−9·7
Medical history of liver disease	31 (1·7%)	6 (3·2%)	10·0	6 (3·3%)	6 (3·3%)	0·0
Medical history of haematological disease (excluding anaemia due to Iron, B12 deficit)	65 (3·5%)	5 (2·7%)	−4·5	7 (3·9%)	5 (2·8%)	−6·5
Medical history of essential hypertension	25 (1·3%)	3 (1·6%)	2·2	3 (1·7%)	3 (1·7%)	0·0
Medical history of kidney renal disease	153 (8·2%)	33 (17·8%)	28·8	27 (15·0%)	28 (15·6%)	1·7
Medical history of STI	297 (16·0%)	50 (27·0%)	27·1	51 (28·3%)	47 (26·1%)	−5·4
Medical history of mental health						
No mental health disorder	862 (46·3%)	64 (34·6%)	−23·9	62 (34·4%)	64 (35·6%)	2·3
Mental health diagnosis with treatment	860 (46·2%)	103 (55·7%)	19·0	99 (55·0%)	99 (55·0%)	0·0
Mental health diagnosis without treatment	138 (7·4%)	18 (9·7%)	7·9	19 (10·6%)	17 (9·4%)	−4·0
Smoking at booking	656 (35·3%)	88 (47·6%)	25·0	86 (47·8%)	84 (46·7%)	−2·3
Illicit drug use at booking	124 (6·7%)	27 (14·6%)	25·8	23 (12·8%)	26 (14·4%)	5·4

This table compares key maternal characteristics between women receiving Waminda care and those receiving standard care, both before and after propensity score matching. Values are presented as counts or percentage (%), with percentage bias included to show covariate balance across cohorts. All data are descriptive and reflect women who gave birth to an Indigenous baby between 1 January 2018 and 30 June 2022.

A logit model was performed to predict the probability of a woman accessing Waminda care, given their profile in terms of the adjusted risk factors, to calculate the propensity score for each birth. The births were matched on a one-to-one basis without replacement, using callipers of the width of 0·2 standard deviation of the logit of the propensity score (calliper 0·34). The balance of the covariates before and after matching were checked using Rubin's B, Rubin's R and the standardised differences. The overall model balance was summarised by Rubin's B (<25%) and Rubin's R (close to 1·0). The standardised difference for each covariate was also reported with less than 10% indicating an adequate balancing in terms of the covariates being assessed (Table 3).

The univariate model was developed for both unmatched and matched samples. Odds ratio (OR) and 95% confidence interval (CI) was calculated to measure the treatment effect between the intervention and standard care groups. To accommodate the 1:1 matching sample, we performed conditional logistic regression for large sample size and exact logistic regression was conducted for any outcomes with a cell value five or less. When compared to the standard care cohort of women who gave birth to an Indigenous baby, the Waminda cohort had a higher proportion of Indigenous mothers - who are known to have a lower socio-economic status and experience higher interventions with poor birth outcomes. To test the potential impact of the Indigenous status of the mother, sensitivity analysis was conducted by excluding non-Indigenous mothers using the same propensity score strategies described above. The analysis was performed with Stata software, version 16·0.

The study did not achieve the required sample size in the intervention cohort,^30^ which may have reduced the statistical power to detect a true effect and increased the risk of a Type II error. This limitation has been taken into account when interpreting the findings, particularly for outcomes with wide confidence intervals. We did not have any loss to follow-up because our inclusion criteria required that women gave birth at the local hospital be included in the analysis, and we had access to a comprehensive dataset for all births within the study period.

### Role of the funding source

The funder of the study had no role in study design, data analysis, data interpretation, or writing of this paper.

## Results

From 1st January 2018 to 30th June 2022, a total of 2234 Aboriginal and Torres Strait Islander babies were born in the Shoalhaven District Memorial Hospital and the Wollongong Hospital. After pre-defined exclusions (n = 189), 185 (9·0%) women received care from Waminda's service and 1860 (91·0%) received standard care. Women in the two cohorts had significant differences in demographics, maternal and obstetric characteristics. Compared to women who received standard care, the Waminda cohort comprised a much higher number of Aboriginal women. In addition, the Waminda cohort faced more complex life stressors with higher rates of teenage pregnancy, sexually transmitted infections, history of renal disease, and higher rates of smoking and illicit drug use. Women in the Waminda cohort were twice as likely to be in the most disadvantaged socioeconomic quintiles, less likely to be married or in de facto relationship, and less likely to have had a previous caesarean section (Table 3). There were no missing data for any of the variables of interest included in the analysis.

**Fig. 3:**
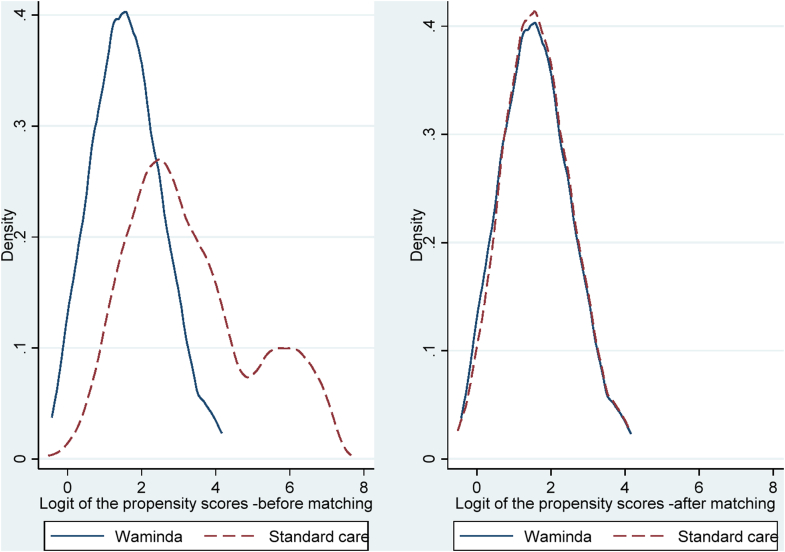
**Density distribution of the logit of the propensity score for Waminda andstandard care cohorts before and after propensity score matching.** This figure compares the density distributions of the logit-transformed propensity scores for women receiving care through Waminda and those receiving standard care, shown separately before and after propensity score matching. Prior to matching, the two groups display clear differences in score distribution, indicating imbalance in observed covariates. After matching, the distributions overlap closely, demonstrating improved covariate balance between the cohorts. Solid lines represent the Waminda group and dashed lines represent the standard care group.

After the 1:1 propensity score matching, all the risk factors listed in Table 3 were balanced with all covariates' standardised difference <10% which suggested that the matched cohorts were highly similar. Rubin's B was reduced from 124·4 before matching to 21·6 after matching and Rubin's R was increased from 0·3 to 0·7 respectively. Density distributions of the logit of the propensity scores for the two cohorts before and after matching showed that after matching the propensity scores distribution were near-identical (Fig. 3). Ninety-seven percent of the women in the Waminda cohort were successfully matched with women in the standard care cohort by their propensity scores which resulted in a 180 equal-sized cohort pairs after matching.

Compared to the standard care cohort, significantly less women from Waminda received their first antenatal assessment within the 1st trimester with a crude OR 0·64 (95% CI 0·45, 0·91), however, after propensity score matching the difference was not statistically significant (OR 0·63, 95% CI 0·38, 1·03). Women with Waminda service had significantly less antenatal visits compared to standard care in both unmatched (OR 0·25, 95% CI 0·16, 0·37) and matched (OR 0·22, 95% CI 0·10, 0·50) cohort, although it is important to note that there were differences in measuring these outcomes (see below). Significantly more women in the Waminda cohort had a normal birth compared to women who had standard care. The odds of having a normal birth was more than 70% higher in both unmatched (OR 1·73, 95% CI 1·25, 2·40) and matched (OR 1·74, 95% CI 1·08, 2·79) cohorts (Table 4).

**Table 4:** Treatment effect of Waminda service on primary outcomes among two cohorts.

	Before propensity score matching			
	Standard care	Waminda	OR (95% CI)	p-value
N	1860	185		
First assessment with health service in the 1st trimester	1518 (81·6%)	137 (74·1%)	0·64 (0·45, 0·91)	0·013
≥5 antenatal visits	1756 (94·4%)	149 (80·5%)	0·25 (0·16, 0·37)	<0·0001
Normal birth	419 (22·5%)	62 (33·5%)	1·73 (1·25, 2·40)	0·001
Preterm	186 (10·0%)	21 (11·4%)	1·15 (0·71, 1·86)	0·561
Healthy baby	1556 (83·7%)	154 (83·2%)	0·97 (0·65, 1·46)	0·885
Exclusive breastfeeding at discharge	1218 (65·5%)	140 (75·7%)	1·64 (1·16, 2·33)	0·006

	After 1:1 propensity score matching			
	Standard care	Waminda	OR (95% CI)	p-value
N	180	180		
First assessment with health service in the 1st trimester	147 (81·7%)	132 (73·3%)	0·63 (0·38, 1·03)	0·065
≥5 antenatal visits	170 (94·4%)	145 (80·6%)	0·22 (0·10, 0·50)	<0·0001
Normal birth	39 (21·7%)	59 (32·8%)	1·74 (1·08, 2·79)	0·022
Preterm	19 (10·6%)	19 (10·6%)	1·00 (0·52, 1·92)	1·000
Healthy baby	155 (86·1%)	151 (83·9%)	0·84 (0·47, 1·50)	0·556
Exclusive breastfeeding at discharge	114 (63·3%)	136 (75·6%)	1·88 (1·16, 3·05)	0·011

Normal birth: term, spontaneous onset, vertex, spontaneous vaginal births, no epidural, no episiotomy.

Healthy baby: liveborn, singleton, term, 2500–4499 g, Apgar score at 5 min ≥ 7.

Variables adjusted in the model: mum Indigenous status, teenager status, SEIFA quintile, marriage status, body mass index, primiparity, previous caesarean section, previous preterm, medical history of heart disease, diabetes, thyroid disease, liver disease, haematological disease, hypertension, renal disease, and sexually transmitted infection, mental health status at booking, smoking and illicit drug usage at booking.

This table shows the comparative effectiveness of Waminda's maternity service versus standard care on key perinatal outcomes, presented both before and after 1:1 propensity score matching. Outcomes are reported as counts (%) with odds ratios (ORs), 95% confidence intervals (CIs), and p-values.

Infants born within Waminda's model of care and standard care had similar odds of being preterm and being a healthy baby. However, Waminda infants had higher odds of being exclusively breastfed at discharge in both unmatched (OR 1·64, 95% CI 1·16, 2·33) and matched (OR 1·88, 95% CI 1·16, 3·05) cohort (Table 4).

The propensity score matched analysis found that Waminda women had significantly higher spontaneous labour onset and spontaneous vaginal birth. Waminda's service significantly reduced the odds of having epidural pain relief by 39% compared to standard care (p = 0·026). We found no difference between these two cohorts in rates of instrumental vaginal births, caesarean sections, 3rd/4th degree tears, episiotomies, low birthweight, and Apgar score at 5 min (Table 5). Twenty-two (1·2%) babies from standard care had Child Protection interventions at hospital discharge compared with none of the Waminda babies, although the difference was not significantly different due to small sample size.

**Table 5:** Treatment effect of Waminda service on secondary outcomes among two cohorts.

	Before propensity score matching			
	Standard care	Waminda	OR (95% CI)	p-value
N	1860	185		
Spontaneous labour onset	705 (37·9%)	93 (50·3%)	1·66 (1·22, 2·24)	0·001
Induction	774 (41·6%)	69 (37·3%)	0·83 (0·61, 1·14)	0·256
Pain management epidural or spinal	1016 (54·6%)	71 (38·4%)	0·52 (0·38, 0·70)	<0·0001
Spontaneous vaginal birth	1071 (57·6%)	133 (71·9%)	1·88 (1·35, 2·63)	0·0001
Instrumental vaginal birth	153 (8·2%)	13 (7·0%)	0·84 (0·47, 1·52)	0·570
Caesarean section	636 (34·2%)	39 (21·1%)	0·51 (0·36, 0·74)	0·0002
3rd/4th degree tear	30 (1·6%)	0 (0·0%)	0·23 (0·00, 1·31)	0·114
Episiotomy	182 (9·8%)	13 (7·0%)	0·70 (0·39, 1·25)	0·226
Low birthweight	158 (8·5%)	12 (6·5%)	0·75 (0·41, 1·37)	0·347
Apgar score at 5 min < 7	48 (2·6%)	5 (2·7%)	1·04 (0·32, 2·65)	1·000

	After 1:1 propensity score matching			
	Standard care	Waminda	OR (95% CI)	p-value
N	180	180		
Spontaneous labour onset	69 (38·3%)	88 (48·9%)	1·51 (1·00, 2·29)	0·050
Induction	81 (45·0%)	69 (38·3%)	0·78 (0·52, 1·16)	0·222
Pain management epidural or spinal	91 (50·6%)	70 (38·9%)	0·61 (0·40, 0·94)	0·026
Spontaneous vaginal birth	110 (61·1%)	129 (71·7%)	1·67 (1·05, 2·68)	0·030
Instrumental vaginal birth	16 (8·9%)	12 (6·7%)	0·75 (0·35, 1·59)	0·451
Caesarean section	54 (30·0%)	39 (21·7%)	0·63 (0·38, 1·03)	0·065
3rd/4th degree tear	0 (0·0%)	0 (0·0%)	–	–
Episiotomy	16 (8·9%)	12 (6·7%)	0·73 (0·34, 1·60)	0·435
Low birthweight	13 (7·2%)	12 (6·7%)	0·92 (0·40, 2·08)	0·835
Apgar score at 5 min < 7	4 (2·2%)	5 (2·8%)	1·25 (0·27, 6·30)	1·000

Variables adjusted in the model: mum Indigenous status, teenager status, SEIFA quintile, marriage status, body mass index, primiparity, previous caesarean section, previous preterm, medical history of heart disease, diabetes, thyroid disease, liver disease, haematological disease, hypertension, renal disease, and sexually transmitted infection, mental health status at booking, smoking and illicit drug usage at booking.

This table presents secondary perinatal outcomes for women receiving Waminda care compared with those receiving standard care, reported before and after 1:1 propensity score matching. Results are shown as counts (%) with odds ratios (ORs), 95% confidence intervals (CIs), and p-values.

Sensitivity analysis examining Aboriginal mothers only did not balance all covariates listed in Table 3 possibly due to lack of high-quality matches with reduced sample size (Table S1, Figure S1).

The most frequent wrap-around services Waminda clients accessed were Waminda General Practitioner (96·2%), Aboriginal Health Practitioner (85·4%), and Registered Nurse (68·6%); on average a woman had 13·6, 4·9 and 2·9 contacts respectively. About 20% of women accessed the Dead or Deadly program (24·9%), Healing Counsellor (20·0%), and crisis support (23·8%), with 9·5, 4·9 and 5·1 contacts on average. More than 10% of women were supported by the Domestic Violence service (12·4%), Child and Parenting service (12·4%), Case management (16·2%), Allied Health (10·3%) and Child safety program (15·1%) with an average of 14·8, 26·6, 15·2, 1·5, and 75·2 contacts respectively. The range of contacts with the child safety program was 1–373 primarily driven by two women who had intensive support with more than 200 visits. Women sought care or services from Balaang Healing (2·7%), Social and Emotional Wellbeing (5·9%), Drug and Alcohol (6·5%), Playgroup (5·4%), Chronic care (3·2%) and Justice Health (2·2%) with 2·6, 22·1, 7·8, 12·7, 7·0 and 12·8 contacts on average (Table 2). As demonstrated in Fig. 2, cross referrals between these programs were experienced and Waminda women were offered intensive wrap-around support from a wide range of programs. Further analysis found that 32 (17·6%) women received services from 2 programs, 87 (47·8%) from 3 programs, 27 (14·8%) from 4 programs, 16 (8·8%) from 5 programs, and 7 (3·9%) from 6 programs.

## Discussion

Culture is the foundation of Waminda's Model of Care© and this is reflected in their commitment to linking culture with education, health and wellbeing in their unique, holistic Aboriginal women-led healthcare service. Many women accessing Waminda's Minga Gudjaga maternity service face complex life stressors including social, emotional and other health issues relating to the ongoing trauma caused by colonisation. Our network analysis showed that more than 90% of women accessing Waminda maternity care received at least one wrap-around service, and some women received services from up to six different programs, with the most frequent service being GP, Aboriginal Health Practitioner and Nurse. More than 20% of women attended a Waminda specific program such as Dead or Deadly, Healing Counsellors and crisis support. Waminda's wrap-around network programs focus on strength-based care addressing the social determinants of health impacting women's lives. This is a key difference between the Aboriginal community-controlled services and the standard care offered through the hospital setting (Table 1). To our knowledge, this is the first time that a network analysis has been performed to quantify the number and type of ante/postnatal contacts with wrap-around programs. As such, this paper serves as an innovative contribution to the literature.

Waminda provide many more services than women would generally have access to in a standard care (hospital or General Practitioner) setting. The wrap-around programs aim to ensure women feel culturally and clinically safe, are provided with high quality holistic care and support to keep families and babies strong and together. Results found a very high uptake of various programs by some women, for example, engaging with Social and Emotional Wellbeing support and programs designed to enhance family preservation (prevent child removal) and protection (ensure child safety). Our data shows that when care provided to women is relevant, culturally safe and acceptable, they engage in services regularly, which is contrary to the common rhetoric about Aboriginal women's engagement with the Australian mainstream setting.^37^

This research site saw partial implementation of the planned service model including: a multi-agency partnership between Waminda as the lead Aboriginal Community Controlled Health Organisation (ACCHO), and the local maternity hospital. Waminda employs a minimum 75% Aboriginal workforce with their service model incorporating Aboriginal community consultation, engagement and governance, a cultural safety framework, and a focus on integrating and coordinating care across primary and tertiary services.^22^

Although the study had aimed to implement continuity of midwifery care through the Midwifery Group Practice, this aim was not realised during the study timeframe due to the service's inability to purchase an insurance product (amongst other issues). Instead, it took many years of advocacy, lobbying, policy briefs, and Ministerial meetings at the Commonwealth and State levels to address the systemic structural barriers preventing this (detailed in another publication^29^).

The study hypothesised that the intervention cohort (Waminda) would see improved maternal and newborn outcomes for women carrying an Aboriginal baby, when compared to a standard care cohort. The primary and secondary outcomes were set after discussions with service providers and aligned with the impactful results seen in the previous BiOC urban setting study in Meanjin (Brisbane), QLD.^22^ The BiOC women receiving the new service were more likely to attend five or more antenatal visits (adjusted odds ratio 1·54, 95% CI 1·13–2·09; p = 0·0064), less likely to have an infant born preterm (0·62, 0·42–0·93; p = 0·019), and more likely to exclusively breastfeed on discharge from hospital (1·34, 1·06–1·70; p = 0·014).^23^ Additionally, secondary outcomes demonstrated fewer epidurals and planned caesarean sections in the intervention cohort, with more women having physiological management of third stage and fewer babies admitted to a neonatal nursery.

At the Waminda rural site, however, we did not see full translation or implementation of the model, and not surprisingly, did not see the same results. This was similar to another translation project, that successfully implemented culturally responsive caseload models for First Nations women in three urban sites and not in the rural site.^38^ Authors noted staffing and resource issues, common to rural areas, may have been a factor. However, perhaps establishing the rural service through a local government area, rather than an Aboriginal Community-Controlled organisation like Waminda, contributed to feasibility. At Waminda, structural barriers took longer to overcome than anticipated, highlighting the well-known barriers to research translation outside of the city. However, they did not prevent progress. Data showed that the Waminda cohort had many more of the risk factors that are normally associated with preterm births and poorer outcomes. Two thirds as many in the cohort were Aboriginal mothers, teenagers, in the most disadvantaged socio-economic quintile and twice as many had no partner, with close to 50% smoking at booking (versus 38%). Additionally, more than half were having treatment for a current mental health condition (53% versus 48%), and double were using illicit drugs at booking (15% versus 8%), and had a history of sexually transmitted infections.

To our surprise, the study did not find Waminda women come earlier (0·63, 95% CI 0·38–1·03, p = 0·065) or attended more antenatal visits (0·22, 95% CI 0·10, 0·50, p < 0·0001), which contrasts with the findings from the urban demonstration site. One possible reason is the different interpretation of ‘gestation of first antenatal visit’ between Waminda midwives and hospital midwives. Post hoc exploration discovered that hospital midwives providing standard care defined it as the gestational age at the first antenatal visit with a general practitioner whereas Waminda midwives interpreted it as the first antenatal visit with a midwife, which typically occurs later and is usually a more comprehensive antenatal visit. This issue has been addressed in subsequent data collections. Waminda now records the first antenatal visit according to the initial provider seen by the woman, whether a general practitioner or a midwife. We had hypothesised that these two quality indicators would drive change based on the theory that continuity of midwifery care, delivered early and frequently, facilitates the development of trusting relationships, disclosure of issues to allow early intervention, and opportunities to modify predictors of preterm birth (e.g., early treatment of infections, reduction of stressors like homelessness and family violence).^39^ An alternative explanation for less antenatal clinical visits is that women were engaging with multiple other providers through the wrap around services, some several times a week, and these may have taken priority. Additionally, women experiencing stressors may face practical barriers to accessing care such as lack of transport and care for other children and fear of disclosure of stressors or challenges that may be perceived to or may lead to child protection involvement. This has been explored in our qualitative work and will be described in forthcoming publications.

Contrary to the urban study, Waminda did not result in significant differences in preterm rates before or after matching; reasons for this require further investigation. Preterm rates have not changed in Australia in 15-years and average 14% for Indigenous babies versus 8% for non-Indigenous babies.^4^ What has been highlighted is the plethora of support services that are available, and taken up by women, when they have access to culturally safe care through an Aboriginal community-led organisation. It is clear, that in line with national recommendations, this is where funding needs to be invested to address the disparity in outcomes for Aboriginal mothers and babies. In the early years of the urban model, when the continuity of midwifery service was implemented before the full model was established, we saw early reduction of preterm births,^40^ that were sustained throughout the study time frame.^23^ This could be the unique contribution of continuity of midwifery care delivered early and often for First Nations families.

Babies in the Waminda cohort were significantly more likely to be exclusively breastfed at discharge compared to the standard care cohort (1·88, 95% CI 1·16–3·05, p = 0·011). This is a priority for the Waminda team and an important outcome, considering the recent WHO reviews which concluded that breastfeeding could improve infant cognitive development, and reduce other chronic diseases in adulthood such as diabetes (type 1 and type 2), obesity, hypertension, cardiovascular disease, hyperlipidaemia and cancer.^41^ Our secondary outcomes highlighted that women in the Waminda cohort are more likely to have spontaneous labour onset, use less epidural pain relief, and experience more spontaneous vaginal births, showing that even without intrapartum care from their known midwife, women who received high quality antenatal continuity of care and education from Waminda were more likely to have a physiological birth, which has positive short- and long-term health implications for the mother and infant, and is known to facilitate optimal newborn transition and support the early initiation of breastfeeding.^42^ These findings are consistent with the results from the urban study.^23^ The breastfeeding rate is likely to be sustained as breastfeeding on hospital discharge is with culturally safe and continued support for Women in Waminda until six weeks post-birth, and after with wrap-around primary health care safety network. Waminda provide comprehensive primary health care services for women and their families throughout the entire life cycle, including child and family health services. As such, there is very little loss of connection during transition from midwifery to child and family health. At the time of data collection, one of the Minga Gudjaga midwives was also the Minga Gudjaga child and family health nurse, therefore she was already a known and trusted care provider. When children require referral to specialist care the Waminda worker arranges for the referral and, if the woman wants support, often accompanies her to the appointment with transport and funding if needed.

Importantly, despite many more risk factors for child removal, and like the BiOC urban study,^25^ the Waminda cohort had less Child Protection interactions from government services at hospital discharge compared to the standard care cohort. Although the numbers are too small to be conclusive this is under further investigation at the site. This may well be due to the wrap around care and support that has been documented here. Full implementation of the Birthing on Country model of care at Waminda is now being evaluated under another prospective trial (ACTRN12623001204639p) and will provide larger numbers to contribute knowledge to this important outcome (reduced Child Safety unborn notifications).

Both the BOOSt and new studies include a process, impact and outcome evaluation with both qualitative and quantitative data items and questions set with the Waminda team at the beginning. Additionally, a program logic was developed to detail the inputs, activities, indicators, impact and short- and long-term outcomes to determine feasibility, acceptability, clinical and cultural safety, effectiveness and cost of this exemplar rural model (#ACTRN 12620 00087 4910). The short-, medium-, and long-term cost–benefit of the Waminda model of care is a critical area of inquiry with a detailed economic modelling to inform both service planning and the broader translation of the model into other contexts under discussion.

Despite a small sample size, our study found that the Aboriginal Community-Controlled Health Organisation Waminda's Minga Gudjaga maternity service significantly improved spontaneous labour onset, spontaneous vaginal birth and reduced epidural pain relief. Babies whose mothers were cared for by Waminda were significantly more likely to be exclusively breastfed at discharge. These results, which will have positive short- and long-term health implications for both mother and infant, are another testimony regarding the effectiveness of Birthing on Country service redesign for Aboriginal and Torres Strait Islander peoples. National implementation of Birthing on Country models of care should be supported by all levels of government in order to redress the ongoing harmful impacts of colonisation and more importantly, return birth to Aboriginal community control; rightly placing Aboriginal health in Aboriginal hands. Funding should be directed to the Aboriginal Community-Controlled Health sector to maximise the chances of success.

## Contributors

Yu Gao was involved in protocol and study design, writing the successful funding proposal, conducted the analysis, interpretation of results and writing of the manuscript. She led the analysis and completed the tables and figures. Sue Kildea was involved in developing the intervention model, led the literature search, protocol development, successful funding application, study design, interpretation and writing of the manuscript. She oversaw data collection and contributed to key decisions in analysis. Rebecca Coddington contributed to the conduct and monitoring of the study, the collection of data, interpretation of results and writing of manuscript. Melanie Briggs, Cleone Wellington, and Faye Worner contributed to the study design, developing the intervention, interpretation and writing of the manuscript. Yvette Roe, Donna Hartz, and Juanita Shertwood provided the Indigenous oversight of the study and were involved in protocol development, study design, writing the successful funding proposal and Indigenous methodology, contributed to writing of the manuscript. Yu Gao and Sue Kildea have directly accessed and verified the data. All authors approved the final manuscript and agreed to submit the manuscript.

## Data sharing statement

The BOOSt study protocol is published and available in an open-access article. The statistical analysis plan will be made available for research purposes upon request to the corresponding author. The deidentified data supporting the conclusion of this article will be available for researchers after approval by the study Steering Committee. For all data-sharing enquiries, please contact the corresponding author at yvette.roe@cdu.edu.au.

## Declaration of interests

The BOOSt study was funded by the National Health Medical Research Council (NHMRC) of Australia Partnership grant (grant 1135125), with cash and in-kind contributions from partner organisations. Yu Gao, Sue Kildea, Yvette Roe were employed by Mater Research Institute and University of Queensland, then Charles Darwin University during the course of the study. Rebecca Coddington, Melanie Briggs, Cleone Worner, Faye Worner were employed by the Waminda, Donna Hartz was employed by the Western Sydney University and Western Sydney Local Health District, and Juanita Sherwood was employed by the University of Technology Sydney.
