# Supplementary material for: Effect of the implementation of a Birthing on Country service at a rural site, Waminda, compared to standard care for First Nations Australians: a prospective, non-randomised, interventional trial
Source: Lancet Reg Health West Pac. 2026 Jan 27;67:101796. doi: 10.1016/j.lanwpc.2025.101796 (PMC12873586; doi:10.1016/j.lanwpc.2025.101796)
Supplement: Tables and Figure [file mmc1.docx]

**Table S1: Maternal characteristics comparisons among two Indigenous mothers cohorts who gave birth during 01/01/2018- 30/06/2022. This table compares key maternal characteristics between Indigenous women receiving Waminda care and Indigenous women receiving standard care between 1 January 2018 and 30 June 2022. Variables include sociodemographic factors, obstetric history, medical conditions, and behaviours at booking. Data are presented as counts (%) or medians (IQR), with p-values indicating group differences.**

| Factor | Non-Waminda Indigenous | Waminda Indigenous | p-value |
| --- | --- | --- | --- |
| N | **1008** | **147** |  |
| Teenager | **91 (9·0%)** | **21 (14·3%)** | **0·044** |
| Socioeconomic status (SEIFA) |  |  | **<0·0001** |
| Quintile 1 (most disadvantaged) | **380 (37·7%)** | **93 (63·3%)** |  |
| Quintile 2 | **363 (36·0%)** | **51 (34·7%)** |  |
| Quintile 3 | **179 (17·8%)** | **2 (1·4%)** |  |
| Quintile 4 | **79 (7·8%)** | **1 (0·7%)** |  |
| Quintile 5 (most advantaged) | **7 (0·7%)** | **0 (0·0%)** |  |
| Married or in de facto | **530 (52·6%)** | **37 (25·2%)** | **<0·0001** |
| BMI, median (IQR) | **25·4 (21·7, 31·2) (n=1008)** | **24·5 (21·1, 28·3) (n=147)** | **0·035** |
| Primiparity | **366 (36·3%)** | **58 (39·5%)** | **0·46** |
| Previous caesarean section | **185 (18·4%)** | **23 (15·6%)** | **0·42** |
| Previous preterm birth | **119 (11·8%)** | **18 (12·2%)** | **0·88** |
| Medical history of Heart disease | **57 (5·7%)** | **10 (6·8%)** | **0·58** |
| Medical history of Diabetes (Type 1 or Type 2) | **6 (0·6%)** | **1 (0·7%)** | **0·90** |
| Medical history of Thyroid disease | **44 (4·4%)** | **8 (5·4%)** | **0·56** |
| Medical history of Liver disease (Viral Hepatitis) | **18 (1·8%)** | **5 (3·4%)** | **0·19** |
| Medical history of Haematological disease (excluding anemia due to Iron, B12 or | **31 (3·1%)** | **5 (3·4%)** | **0·83** |
| Medical history of Essential hypertension | **11 (1·1%)** | **3 (2·0%)** | **0·33** |
| Medical history of Kidney renal disease | **91 (9·0%)** | **31 (21·1%)** | **<0·001** |
| Medical history of STI | **176 (1705%)** | **40 (27·2%)** | **0·005** |
| Medical history of Mental health conditions treatment |  |  | **0·061** |
| No Mental Health Disorder | **445 (44·1%)** | **52 (35·4%)** |  |
| Mental Health diagnosis with treatment | **488 (48·4%)** | **78 (53·1%)** |  |
| Mental Health diagnosis without treatment | **75 (7·4%)** | **17 (11·6%)** |  |
| Smoking at booking | **388 (38·5%)** | **70 (47·6%)** | **0·035** |
| Illicit drug user at booking | **77 (7·6%)** | **22 (15·0%)** | **0·003** |

**Table S2: Consolidated criteria for strengthening reporting of health research involving Indigenous peoples (CONSIDER) checklist. This table outlines how the study meets the CONSIDER criteria across governance, priorities, relationships, methods, participation, capacity building, analysis, and dissemination. Summary notes describe the Indigenous-led governance, ethical processes, community involvement, and culturally informed research approach underpinning the project.**

| **Item Checklist Item**   - **much of this is covered in the paper but we have additional information below.** | |
| --- | --- |
| **Governance** | |
| **1.** | **Describe partnership agreements between the research institution and Indigenous-governing organization for the research, (e.g., Informal agreements through to MOU (Memorandum of Understanding) or MOA (Memorandum of Agreement)).**   - **A joint funding agreement was successful and this drove the development of the research study with an overarching Steering Committee and MIA (multi-institutional agreement) between the Molly Institute and Waminda.** |
| **2.** | **Describe accountability and review mechanisms within the partnership agreement that addresses harm minimization.**   - **The national recommendations for conducting research with Aboriginal and Torres Strait Islander peoples were met.** - **Regular Steering Committee meetings, chaired by Waminda, oversaw the project with regular research committee meetings sitting underneath and reporting up.** - **Additionally, the research team reported regularly verbally, and in writing, to the Waminda research committee.** - **The research was driven by a Participatory Action Research approach led by Waminda with the research team being responsive to Waminda’s goals and wishes.** |
| **3.** | **Specify how the research partnership agreement includes protection of Indigenous intellectual property and knowledge arising from the research, including financial and intellectual benefits generated (e.g., development of traditional medicines for commercial purposes or supporting the Indigenous community to develop commercialization proposals generated from the research).**   - **Waminda’s Intellectual and cultural property is protected through a signed contract between the partners.** - **Waminda sought legal advice and submitted additional paragraphs to the multi-institutional agreement to ensure this was the case.** - **Waminda have contributed to all research outputs arising from this project and have reviewed the final manuscript to ensure it accurately reflects their service.** |
| **Prioritization** | |
| **4.** | **Explain how the research aims emerged from priorities identified by either Indigenous stakeholders, governing bodies, funders, non-government organization(s), stakeholders, consumers, and empirical evidence**   - **As noted above - the research was driven by community, with the initial stage of the project including community consultations driven by Waminda to better understand the needs and desires of the local Aboriginal community in relation to maternity care.** - **This project was also in alignment with the National Maternity Service Action Plan to develop and evaluate Birthing on Country Services in Rural Australia and met multiple other Australian Government Closing the gap priorities and Funding Body (NHMRC) guidelines.** |
| **Relationships (Indigenous stakeholders/participants and Research team).**   - **The research team were approached by Waminda to discuss the potential of a collaboration. This led to a joint funding application that was developed and submitted together (see published protocol) and this was successful. This process took several years before funding was received and the project started.** | |
| **5.** | **Specify measures that adhere and honor Indigenous ethical guidelines, processes, and approvals for all relevant Indigenous stakeholders, recognizing that multiple Indigenous partners may be involved, e.g., Indigenous ethics committee approval, regional/national ethics approval processes.**   - **The overarching governance and ethics oversight and review ensured this occurred appropriately.** - **The project was developed in collaboration with Waminda and presented to the Waminda research committee for consideration and approval.** - **Ethics applications were submitted to the Aboriginal Health and Medical Research Council along with the joint University of Wollongong - Illawarra Shoalhaven Local Health District Human Research Ethics Committee.** |
| **6.** | **Report how Indigenous stakeholders were involved in the research processes (i.e., research design, funding, implementation, analysis, dissemination/recruitment).**   - **See section 4 above** |
| **7.** | **Describe the expertise of the research team in Indigenous health and research.**   - **Melanie Briggs is an Aboriginal Midwife and the Senior Manager of Birthing on Country at Waminda where she has worked for over 10 years. Melanie has been instrumental in the establishment of Birthing on Country at Waminda and is recognised as a national leader on Maternity Service Re-design for Birthing on Country.** - **Cleone Wellington is a member of Waminda's Chief Executive Leadership Team and the Cultural Lead for Birthing on Country. Cleone has been instrumental in the establishment of Birthing on Country at Waminda and has over 15 years ACCHO experience.** - **Faye Worner is the Chief Operations Officer at Waminda and has been instrumental in the establishment of Birthing on Country at Waminda. Faye has over 18 years ACCHO experience.** - **Rebecca Coddington is a Midwifery Academic with 15 years research experience and holds the position of Birthing on Country Research and Implementation Coordinator at Waminda.** - **Yvette Roe is an Aboriginal scholar with more than 25 years experience working in the Indigenous health** - **Sue Kildea is a non-Indigenous researcher with more than 30 years experiences working in the Indigenous health** - **Donna Hartz is an Aboriginal scholar, with over 40 years experience as a midwife, nurse, educator, lecturer, manager, and researcher.** - **Juanita Sherwood is an Aboriginal Professor having worked for over 35 years as a nurse, teacher, lecturer, and researcher to change health and education outcomes for First Nations peoples** - **Yu Gao is a non-Indigenous researcher with more than 18 years experiences working in the Indigenous health, specialising in quantitative analysis, health economics and data support, reporting and analysiing for Indigenous-led evaluations and service redesigns** |
| **Methodologies** | |
| **8.** | **Describe the methodological approach of the research including a rationale of methods used and implication for Indigenous stakeholders, e.g., privacy and confidentiality (individual and collective)**   - **This is covered thoroughly in the methods section of both the research protocol and this paper.** |
| **9.** | **Describe how the research methodology incorporated consideration of the physical, social, economic and cultural environment of the participants and prospective participants. (e.g., impacts of colonization, racism, and social justice). As well as Indigenous worldviews.**   - **This was ensured through the overarching governance being driven by the community controlled organisation itself and the use of Participatory Action Research (PAR).** - **PAR is a collaborative approach where researchers and participants work together to identify issues, generate knowledge, and implement solutions. PAR prioritises Indigenous ways of knowing, self-determination, and cultural safety. Methods were adapted to ensure respect for Country, language, kinship, and community governance. Community members were engaged as co-researchers, guiding the research agenda, shaping data collection (e.g. yarning, storytelling, group discussions), and determining how findings were shared. This approach supports empowerment, ensures findings are relevant and beneficial, and helps address historical power imbalances in research.** |
| **Participation** | |
| **10.** | **Specify how individual and collective consent was sought to conduct future analysis on collected samples and data (e.g., additional secondary analyses; third-parties accessing samples (genetic, tissue, blood) for further analyses).**   - **No samples (genetic, tissue, blood) were collected.** - **All research questions and analysis were conducted only after the Waminda research committee had approved it.** - **Participant Information Sheets thoroughly detailed how data would be used. Data will not be used for any other purposes (e.g. secondary analysis).** |
| **11.** | **Described how the resource demands (current and future) placed on Indigenous participants and communities involved in the research were identified and agreed upon including any resourcing for participation, knowledge, and expertise**   - **As described above – this was all determined by the Waminda team. Over the past decade, The Molly Institute have invested in relationship building with Waminda and ensured a process of continuous consent in relation to our research collaboration.** - **Waminda were provided with funding to employ a Project Officer and community researcher who completed work on this project. Furthermore, a post-doctoral researcher was employed by the Molly Institute and based at Waminda.** |
| **12.** | **Specify how biological tissue and other samples including data were stored, explaining the processes of removal from traditional lands, if done, and of disposal.**   - **No biological tissue or other samples were collected. Data was stored on the university secured SharePoint with 24/7 back up, and with limited access by relevant research staff only.** |
| **Capacity** | |
| **13.** | **Explain how the research supported the development and maintenance of Indigenous research capacity (e.g., specific funding of Indigenous researchers).**   - **Funding was available for First Nations community researchers to work alongside the research team.** |
| **14.** | **Discuss how the research team undertook professional development opportunities to develop the capacity to partner with Indigenous stakeholders?**   - **Onsite researchers were involved in regular critical reflection by participating in the Waminda Imperfect Allies program.** - **Waminda also provided a 2-day Imperfect Allies workshop for the Research Team and employees of the Molly Wardaguga Research Institute.** |
| **Analysis and interpretation** | |
| **15.** | **Specify how the research analysis and reporting supported critical inquiry and a strength-based approach that was inclusive of Indigenous values.**   - **The PAR approach, led by the Waminda team, ensured that regular reflection occurred, was strength based and inclusive of Indigenous values.** - **Analysis was undertaken collaboratively with community members to ensure interpretations reflected lived experience, cultural values, and collective priorities. This process supports critical inquiry by challenging deficit-based narratives, exposing structural inequities, and positioning Aboriginal voices at the centre of knowledge production. Data were interpreted through Indigenous frameworks—such as relationality, reciprocity, and connection to Country—so that findings are grounded in the priorities of community.** - **Reporting takes a strength-based approach, highlighting resilience, cultural continuity, and community-led solutions rather than focusing solely on ‘problems’ or deficit-based thinking.** |
| **Dissemination** | |
| **16.** | **Describe the dissemination of the research findings to relevant Indigenous governing bodies and peoples.**   - **Dissemination only occurs after it has been endorsed by Waminda and most is led by the Waminda social marketing team.** - **It is anticipated that the results from this research will further enhance support of Waminda’s innovative model of care and will be used to lobby government bodies for future funding and health system redesign.** |
| **17.** | **Discuss the process for knowledge translation and implementation to support Indigenous advancement (e.g., research capacity, policy, investment).**   - **This is already occurring at a national level through Waminda’s and the Molly Institutes involvement in the National Road Map for Birthing on Country, through multiple publications that are coming out from this study and joint conference and other presentations.** - **Waminda is a partner in developing the Birthing on Country Toolkit, a series of online modules designed to provide step-by-step advice on how to set up a Birthing on Country model of care.** - **The Molly Institute continue to work in partnership with Waminda on the next phase of research, which involves capacity building for community researchers and greater community control of research design, implementation and outputs.** |

**Figure S1: Standardized mean difference (%) for unmatched and matched cohort between Waminda and Standard Care cohort-Indigenous mothers only. This figure displays the mean difference (%) for each covariate in the Indigenous-only cohort, comparing Waminda and standard-care groups before and after propensity score matching. Circles represent unmatched differences and crosses represent matched differences.**
